# Imagine All The Synchrony: The effects of actual and imagined synchronous walking on attitudes towards marginalised groups

**DOI:** 10.1371/journal.pone.0216585

**Published:** 2019-05-14

**Authors:** Gray Atherton, Natalie Sebanz, Liam Cross

**Affiliations:** 1 Department of Psychology, Health and Learning Sciences, University of Houston, Houston, United States of America; 2 Department of Cognitive Science, Central European University, Budapest, Hungary; 3 Department of Psychology, School of Science and Technology, Sunway University, Selangor, Malaysia; University of Bologna, ITALY

## Abstract

Stereotyping is a pervasive societal problem that impacts not only minority groups but subserves individuals who perpetuate stereotypes, leading to greater distance between groups. Social contact interventions have been shown to reduce prejudice and stereotyping, but optimal contact conditions between groups are often out of reach in day to day life. Therefore, we investigated the effects of a synchronous walking intervention, a non-verbal embodied approach to intergroup contact that may reduce the need for optimal contact conditions. We studied attitude change towards the Roma group in Hungary following actual and imagined walking, both in a coordinated and uncoordinated manner. Results showed that coordinated walking, both imagined and in vivo, led to explicit and implicit reductions in prejudice and stereotyping towards both the Roma individual and the wider Roma social group. This suggests that coordinated movement could be a valuable addition to current approaches towards prejudice reduction.

## Introduction

Throughout our daily lives, we are prone to using a number of heuristics to quickly differentiate and better understand persons unknown. One of these heuristics is to categorize individuals on the basis of their external attributes, such as their appearance, language, mannerisms, cultural identities, professions, religions etc. It is thought people then form expectations of individuals on the basis of these categorical attributes (i.e. ‘he is Jewish so he will be good with money’ or ‘she is a mother so she will be sympathetic’). Of course, many expectations may also stem from negative attributes of social categories (i.e. ‘he is Jewish so he is stingy’ or ‘she is a mother so she is less career-driven’). Both positive and negative categorical attributes are forms of stereotypes (attributions to an individual made on the basis of their group membership). When negative stereotypes about a certain group become pervasive in society, it may be more accep1 for those in the majority to express overt prejudice towards minority groups, leading to persecutions [1].

As stereotypes are socially constructed, they also have the power to be manipulated, as argued recently by Fiske [2] who asserts that stereotyping is essentially a form of control. On the individual level, people in the minority are pressured by those in the majority to either behave in ways that counter negative group stereotypes or, conversely, in ways that are in line with what may be positive group stereotypes. Stereotyping can also be used as a tool to control the majority. For instance, when people are primed with negative stereotypic portrayals of certain groups they have been shown to generalize these stereotypes to similar individuals over contexts and time [3, 4], meaning that stereotypes can be purposefully used to mobilize others against certain groups. A relevant example of the mobilizing power of stereotype priming is when media portrayals of stereotypical group members are able to influence political outcomes [5]. A number of studies show the effect that negative stereotyping has had on public policy, in particular mobilizing voters on issues relating to gay rights [6], poverty [7], immigration [8], and even influencing election outcomes by perpetuating gender stereotypes [9, 10]. In this way, stereotypes can be seen as tools to not only control those who are the victims of stereotyping, but also as a way to control those who can be roused by the perpetuation of certain stereotypes.

One recent example of public policy and election outcomes being influenced by stereotype activation can be found in Hungary with regards to people of Roma origin, a group which comprises approximately 5–6% of the population, and 10–12% of the young adolescent population [11]. Recent research links negative media coverage of Hungarian Roma to the growing acceptance of anti-Roma rhetoric within mainstream Hungarian political discourse [12]. For instance, in 2009 the Hungarian Jobbik party won 14.8% of votes for the European Parliament election, and 16.7% of the votes in the 2010 national election, with high support among Hungarian youth [13]. It has been suggested that some of their success was driven by removing taboo from anti-Roma sentiments, and repeatedly voicing anti-Roma views [14, 15]. During the period marking the rise of the Jobbik party (2002–2009), surveys indicated that prejudice against the Roma rose to 43% among youth (ages 15–30), and that 47% of youth reported that they would reject having a Roma desk mate [14]. Analysis of Hungarian school textbooks has revealed that Roma do not figure prominently, and when they do they conform to negative stereotypes [16]. A survey of Hungarian adults indicated that 88% believed that a typical Hungarian parent would not allow their child to meet with a Roma playmate [17]. The rise of anti-Roma sentiment in Hungary, particularly among youth, indicates that the current reports of elevated rates of Roma directed hate speech [18] and hate crimes [19] may not abate without direct intervention.

There are several approaches to reducing prejudice towards minority groups. One avenue is to enact public policies that support initiatives beneficial for given minorities and reduce inequality with regards to access to education and distribution of wealth and power. However, those who hold political power will often perpetuate negative stereotypes in an effort to advance political agendas that play on populist fears of minority cultures supplanting the traditional native culture. Another avenue for overcoming intergroup prejudice can arise through changes within individuals in the majority or dominant culture who may then work to create change.

One of the most effective approaches to reducing prejudice between groups has been putting individual group members in situations of direct inter-group contact [20]. A central element of inter-group contact theory is that it should allow members to reappraise their faulty thinking and form important social bonds outside of their specific group. Initially, Allport [20] ascribed the reduction in prejudice following inter-group contact to be mediated by cognitive mechanisms such as knowledge of inter-group commonalities. However, more recently literature has shown that the affective rather than cognitive dimensions of contact are the strongest determinant of prejudice reduction [21]. Specifically, individuals who have limited contact with a member of an out-group experience significantly more anxiety at the prospect of inter-group interactions. Through experiencing contact with an out-group member, anxiety about future meetings can be reduced, which leads to reduction in prejudice [22]. Similarly, research has found that contact promotes empathy and perspective taking towards out-group members, which also mediates prejudice reduction [23].

Contact need not only occur within the realms of traditional, verbal interactions. Research investigating non-verbal interactions also demonstrates cognitive and affective changes that can lead to positive inter-group relations [24, 25]. Using the rubber-hand illusion, a reduction in negative implicit attitudes has been shown in participants who experienced ownership over a hand that looked as if it belonged to an out-group member [26, 27]. Research on motoric synchronicity between partners indicates that moving in time with another person leads to greater affiliation [28, 29] and may lead to a re-categorization of the other as a common group member [30, 31, 32]

Approaching stereotype reduction through a non-verbal, embodied method such as motoric synchrony poses several advantages over more verbally driven contact techniques. For one, non-verbal embodied methods may be increasingly accessible to participating individuals as they do not require the same requirements for social engagement between participants, such as sharing a common language. They also may allow more precise research into the underlying mechanisms of contact that lead to prejudice reduction, as traditional contact research relies upon a number of optimal conditions that promote pro-social effects [20]. For instance, researchers suggest that optimal contact conditions are ones that allow inter-group contact to occur across a variety of settings, be regular and frequent, occur between people of equal status who are particularly representative of their group, and lead to genuine friendship formation [33].

Critics of inter-group contact have focused on the infeasibility of optimal contact conditions [34]. In particular, there is evidence showing that naturally occurring inter-group contact is more often occasional and superficial rather than optimal [35, 36], and homophily, or the tendency to form social networks with similar others, is most pervasive in friendship groups [37]. Thus, while contact interventions work in controlled settings, they pose challenges with regards to real-world implementation. More importantly, deciding whether optimal contact conditions have been met is a somewhat subjective process. As discussed by [34], participants’ constructions of their contact conditions are complex, and thus difficult to capture and operationalize. This creates a need for more simplified contact conditions in an effort to better explore underlying mechanisms that make contact effective.

As previously discussed, one barrier to typical intergroup contact is its dependence on verbal interactions. On top of requiring participants to speak a common language and have common cultural reference points, participants often fear that they will say something culturally insensitive or stereotype confirming, which can influence the perceived success of the interaction and a subsequent interest in further out-group contact [38]. For these reasons, a non-verbal embodied intervention may have an advantage over traditional contact paradigms as it does not require a common language or shared cultural references. Furthermore, as it is non-verbal it may reduce the chance that participants will negatively self-appraise their social performance with an out-group member, which weakens the benefits of the intervention. Furthermore engaging in coordinated actions such as synchronous walking, chanting and music making naturally occur across cultural and temporal lines [39]. We therefore offer a naturalistic alternative to traditional intergroup contact. The approach used here was to employ a non-verbal synchronous movement task in which Non-Roma Hungarian participants either walked synchronously or asynchronously with a member of the Roma community, in order to ascertain changes in their attitudes towards both that individual and the minority group.

## Method

### Participants

Seventy people participated in this study (45 females, 24 males, 1 other, M_age_ = 24.33yr, SD_age_ = 5.16). All participants were recruited from the Central European University’s (CEU) participant database and consisted of both CEU students, students from other local universities, and non-student populations. All participants identified as being from a Non-Roma Hungarian background, were Hungarian speaking, and were naïve to the aims of the study. Participants were compensated with a 2000 HUF (roughly 7 euros) gift voucher. The experiment was approved by the (EPKEB) United Ethical Review Board for Research in Psychology and took approximately 40 minutes to complete.

### Design and procedure

This experiment employed a between groups design, and participants were tested individually in separate sessions. First, participants completed measures of explicit attitudes, overlap and empathy towards Non-Roma Hungarian and Roma people (see materials section, for a detailed description of all materials) online through the Survey Monkey platform. One week later participants came in person to the lab where they were matched across conditions using their initial attitude scores to ensure conditions were roughly equal. In the lab, participants first completed a custom Implicit Association Test (IAT) [40]. Following this, participants were introduced to a Roma confederate whom they performed a walking task with. The single confederate was a male in his mid 20s from the Roma community. He was unknown to participants, unaware of the research hypothesis and acted as a naïve participant. The group distinction (Roma / Non-Roma Hungarian) was made clear to participants using a primed introduction, whereby the confederate was introduced as another participant (“as you know this study is about how people act with members of the same or different background to you. One of you is Roma, and one is non-Roma Hungarian”). The participant and confederate were then asked to introduce themselves to one another by name and shake hands.

Participants then took part in the walking task, whereby they either walked in a Synchronous or Uncoordinated way along with the confederate for three minutes. People walked laps of a long room side by side (1m apart). A grid was marked out on the floor measuring 12 meters in length, with two straight lines for the participants to walk along, and 21 stepping points between the end points (each stepping point was one ½ meter apart). Participants were asked to step on the markers and stepped 22 times each lap. In the experimental condition (Synchronous) participants were asked to walk synchronously, so as to land their footsteps on the stepping points at the same time. Their pace was initially primed by a metronome set at 85 BPM. Participants were instructed when to begin and heard this metronome for the first 20 seconds they were walking. Upon reaching the end points of the room participants were instructed to pivot with their left foot and continue walking in the opposite direction. After a 5-minute training period participants performed 6 laps of the room taking about 3 minutes. In the Uncoordinated condition participants did exactly the same except instead of walking together at the same pace they were asked to walk to their own pre-specified paces, one faster, one slower. Participants were primed for the first 20 seconds with different metronomes (75 / 90 BPM), played individually through headphones. Whether the participant walked slower or faster than the confederate was counterbalanced across trials. In all conditions the confederate was trained to walk at the correct pace to the required BPM, and instructed not to talk to the participant. All walking sessions were video recorded.

After people had completed the walking task they were taken back to another room where they completed measures of affiliation, overlap and empathy towards the confederate and second copies of the IAT, as well as explicit attitudes measures (identical to those they had previously completed). Participants also rated how difficult they found the walking task and how coordinated they thought they had been walking. Following this, participants were debriefed. All instructions and measures were given in Hungarian, translated from English and checked by three bilingual members of staff at the Cognitive Science department at CEU.

### Materials

All measures were completed on a computer and (except for the overlap measure) were responded to on a slider scale weighted by appropriate anchor points (i.e. not at all–very) generating a score from 0–100.

#### Affiliation, overlap and empathy

The affiliation measures consisted of 5 questions measuring how close, similar, connected participants felt to their partner, how much they felt they were on the same team, and how much they wanted to see them again. These measures were taken from those used in similar research on motoric synchronicity [41, 42, 43, 44]. The measure of overlap was the inclusion of self in other measure [45], where participants were asked to rate which of 7 different images of overlapping circles (from completely separate to completely overlapping) best depicted their relationship with the other participant. Separate items also assessed overlap with the confederate, Roma people as a whole, and non-Roma Hungarians. Empathy was measured using the Felt Understanding Measure [46], a measure developed to assess empathy towards members of minority groups. It consisted of five questions assessing how well participants felt they understood the other participant, could feel what they felt, could walk in their shoes, could connect to them, and how much empathy they had for them. Separate items assessed empathy with Roma people as a whole, and non-Roma Hungarians.

#### Explicit attitudes

Attitudes and stereotypes towards each group were assessed using a selection of questions from the Prejudicial Attitudes Measure [47] and questions used by the Harvard explicit study [48]. These specific questions were chosen after consultation with members of the Roma studies program at CEU to determine their appropriateness to measure prejudice and stereotypes towards these groups (A. Kovács, personal communication, February 2018). We combined these questions into two measures, one of which was a directed attitudes measure. This consisted of eleven questions assessing common stereotypes/attitudes, such as how aggressive, lazy, hardworking and determined to succeed a given group is considered to be, how willing one would be to have a member of a given group as their boss, sexual partner, or to have them join one’s close family in marriage, how much sympathy one has for a given group, how different one feels such individuals are from one’s family members, and finally whether one feels a given group receives too much in government support or whether politicians care too much about said group. These questions were presented twice, once with Roma as the target of evaluations and once with non-Roma Hungarians. Additionally, a further 5 questions were used to form a comparison measure. This asked participants to assess how different Roma are from non-Roma Hungarians, whether Roma teach their children different values, how similar Roma are, how much participants believe Roma come from a less able ethnic group, and whether they have jobs that ‘Non-Roma Hungarians’ should have. Relevant items were reversed scored so that in all cases larger scores indicated more negative attitudes or larger differences between the two groups.

#### Implicit attitudes

Implicit attitudes were assessed using an IAT [49], which measures implicit or automatically activated evaluations. In order to do this, participants are presented with a pair of targets (Roma and Hungarian) and a number of attributes (words falling into the categories “good” and “bad”). Each target is then paired with the positive or negative attribute, and the speed at which participants accurately match the target and attribute are measured. Faster and more accurate responses for a particular target/attribute pairing show a strong association between that pairing. In this task, the target variables were five pictures of traditionally dressed Roma people, five pictures of traditionally dressed Hungarian people, and one picture of each group’s respective flag. The attributes consisted of 6 positive words, and 6 negative words. The use of pictorial rather than verbal stimuli to depict the target categories was chosen as research has shown that pictures are more emotionally evocative than words [50, 51], and pictures have been shown to more directly capture the experiences associated with the images they represent [52]. The IAT consisted of 7 blocks. Participants were instructed to press the ‘e’ key on the keyboard when the picture or word matched the target variable on the left, and the ‘I’ key when the term matched the target variable on the right. Incorrect responses presented participants with a black screen and a red ‘x,’ and then prompted them to press the correct key. Please see [49] for a more detailed description of the IAT procedure.

### Statistical methods

All data was analysed using between groups or one sample tests, in SPSS with a .05 threshold for significance. Cronbach’s alphas were also calculated on pre-scores from all participants in SPSS. Pre-scores were used as participants in both conditions had had equal experiences in regards to the study at this point. Distributions were assessed for normality using Shapiro-Wilk tests, and Mann-Whitney U and one sample Wilcoxon signed-rank tests were used in lieu of independent samples t-tests wherever the assumption of normality was violated for between group tests. For parametric tests where equality of variance was violated equal variances not assumed tests are reported. For one sample tests, t tests were used when normality was not violated, one sample Wilcoxon signed rank tests were used when it was, and the hypothesised means/medians in these cases were 0.

## Results

### Manipulation checks

We first checked that participants in each condition were performing the walking task adequately. Each walker’s right foot steps were timestamped by one of the researchers using ELAN software to measure when the foot hit the floor. The proportion of steps that were landed within 500ms was then calculated. Those in the Synchronous condition landed significantly more steps within 500 ms of each other than those in the Uncoordinated condition (u = 1225.5, p < .001, r = .92). We also used two check questions measuring task difficulty and perceived coordination. While those in the Synchronous condition did report more perceived coordination than those in the Uncoordinated condition (u = 1155.5, p < .001, r = .76), task difficulty did not significantly differ between the two conditions, (u = 1176.0, p = .434, r = .09). Please see Table 1 for descriptive statistics. We also checked whether there was any difference in reported task difficulty amongst those who performed the slow (n = 18) or fast (n = 17) version of the uncoordinated walking task. There was no difference in difficulty amongst those who performed these versions of the uncoordinated task (t(33) = .198 p = .844, d = .1). These results indicated that our walking manipulation had created the desired context in order for us to interpret the below results.

**Table 1:** Descriptive statistics for manipulation check questions.

		Mean (SD)	Median (range)
Perceived Coordination	Synchronous	87.49 (10.69)	91.3(59–100)
	Uncoordinated	38.69 (25.94)	39 (0–100)
Task Difficulty	Synchronous	29.83 (25.2)	22 (0–89)
	Uncoordinated	35.0 (26.95)	37 (0–100)
Steps within 500 ms	Synchronous	.979 (.03)	.98 (.82–1)
	Uncoordinated	.352 (.06)	.36 (.24-.44)

### Affiliation, overlap and empathy towards the confederate

**Fig 1:**
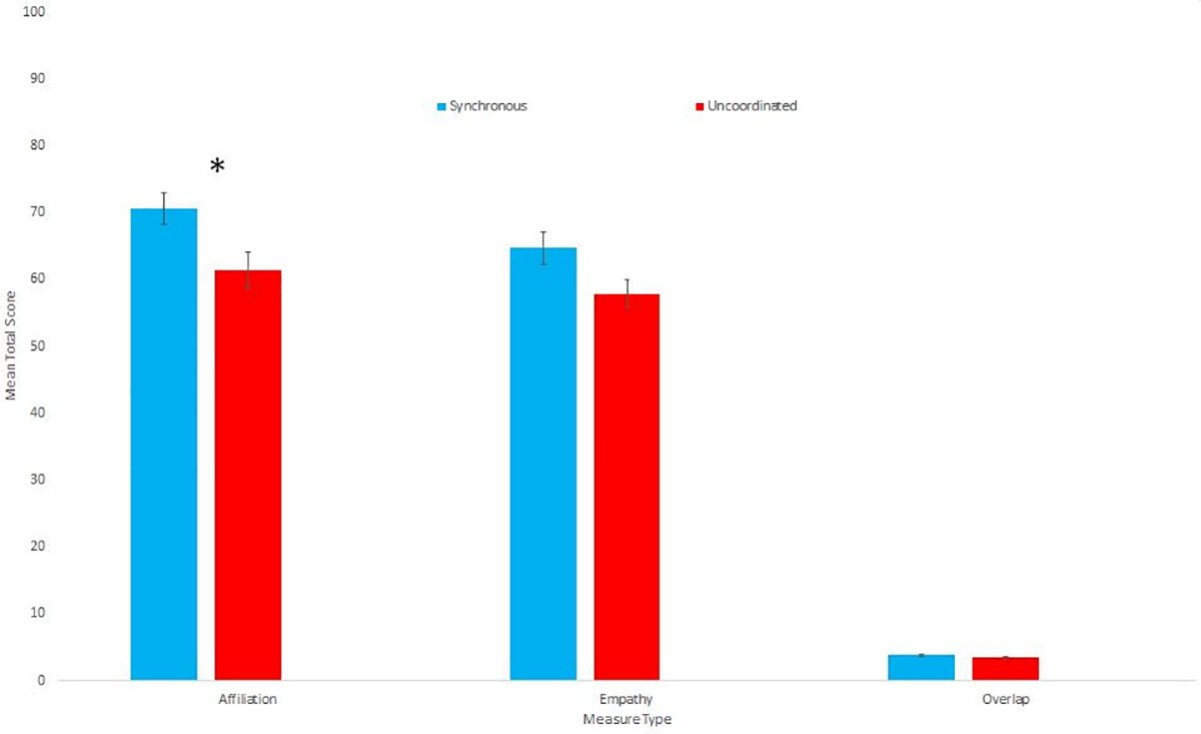
Means and standard errors for the Affiliation, Empathy and Overlap ratings towards the confederate.

We then explored whether there was any difference in ratings of affiliation, overlap or empathy towards the confederate between conditions. We first combined the five affiliation items into a composite score by taking the mean (Cronbach’s a = .859). Those who had walked in a synchronous way reported feeling significantly more affiliated towards the confederate than those who had walked in an uncoordinated way (t(68) = 2.58, p = .012, d = .62). We also combined the five empathy items in the same way (Cronbach’s a = .794). Those in the Synchronous condition reported more empathy towards the confederate than those in the Uncoordinated condition, but this difference did not reach significance (u = 761.5, p = .08 r = 0.21). While those in the Synchronous condition reported more overlap towards the confederate than those in the Uncoordinated condition, this difference was not significant (u = 703.0, p = .274 r = 0.13). Table 2 gives the descriptive statistics for all of the above measures, and Fig 1 shows the means and standard errors.

**Table 2:** Descriptive statistics for the total Affiliation, Empathy and Overlap ratings towards the confederate.

		Mean (SD)	Median (range)
Affiliation	Synchronous	70.58 (13.74)	68.0 (43.0–100)
	Uncoordinated	61.37 (16.07)	62.4 (10.2–95.2)
Empathy	Synchronous	64.63 (14.32)	63.0 (39.2–100)
	Uncoordinated	57.79 (12.23)	58.4 (17.0.– 79.6)
Overlap	Synchronous	3.74 (1.34)	3.71 (2–7)
	Uncoordinated	3.37 (1.09)	3.32 (2–5)

### Empathy and overlap towards Hungarian vs Roma groups

**Fig 2:**
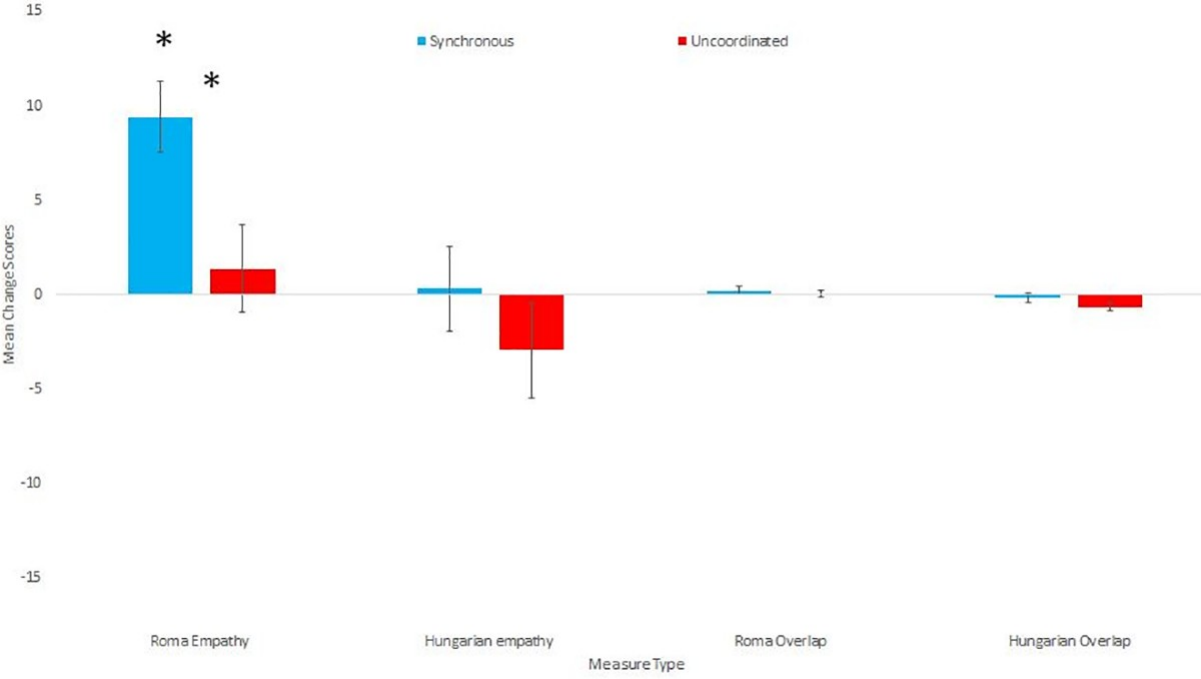
Means and standard errors for the Overlap and Empathy change scores towards Hungarians and Roma.

Both Empathy and Overlap were measured separately towards Roma and Non-Roma Hungarians, both a week before and directly after the experimental manipulation. We created overlap change scores separately for each measure, by subtracting before from after scores. There was no significant difference in overlap change scores between the conditions for either Roma overlap (u = .655.5, p = .60 r = 0.07) or Non-Roma Hungarian overlap (u = 726.5, p = .157 r = 0.17). For empathy scores (Roma empathy a = .87, Non-Roma Hungarian a = .846), we created composite change scores by subtracting each before score from the after score and taking the average of the change scores. There was significantly greater positive empathy changes amongst those who had participated in the Synchronous than in the Uncoordinated condition for Roma empathy, (u = 822.0, p = .014 r = 0.29) but not for the Non-Roma Hungarian empathy (u = .683.5, p = .404 r = 0.1). Please see Table 3 for descriptive statistics. We then explored which of these empathy change scores significantly differed from 0 separately for each condition. Only the Roma empathy change score in the Synchronous condition significantly differed from 0 (Z = 564, p < .001, r = 0.69). None of the Synchronous Non-Roma Hungarian empathy (Z = 351, p = .555, r = 0.10), uncoordinated Roma empathy (t(34) = 0.585, p = .562, d = .099) or the uncoordinated Non-Roma Hungarian empathy (Z = 264.5, p = .408, r = 0.14) change scores differed significantly from 0. All descriptive statistics for both the group level overlap and empathy measures can be found in Table 3. Fig 2 shows the means and standard errors for group level overlap and empathy measures.

**Table 3:** Descriptive statistics for the overlap and empathy change scores towards Hungarians and Roma.

		Mean (SD)	Median (range)
Roma Overlap	Synchronous	0.20 (1.30)	0.19 (-2.0–3.0)
	Uncoordinated	0.03 (1.12)	0.05 (-2.0–3.0)
Hungarian Overlap	Synchronous	-0.17 (1.38)	-0.10 (-4.0–3.0)
	Uncoordinated	-0.66 (1.28)	-.44 (-4.0–1.0)
Roma Empathy	Synchronous	9.38 (11.15)	7.6 (-5.6–42.8)
	Uncoordinated	1.37 (13.86)	0.8 (-32.4–29.4)
Hungarian Empathy	Synchronous	0.30 (13.32)	1.2 (-51.6–28.8)
	Uncoordinated	-2.94 (14.91)	0.2 (-54.6–29.2)

### Explicit attitudes towards Hungarian vs Roma groups

The Cronbach’s alpha for the five comparison questions was only .549, and deleting any items did not improve it. Exploratory Factor analysis showed all items loaded above .5 on one factor, and only one item loaded (marginally) higher on a second factor. Therefore, we made a single composite change score of all five items as originally planned. Those in the Synchronous condition reported larger negative composite change scores (indicating they saw less distinction between the groups) than those in the Uncoordinated condition (t(68) = 2.29, p = .026, d = .55). We then analysed which of these change scores differed from 0 separately for each condition using one sample t-tests. Only the change scores of those in the Synchronous condition significantly differed from 0 (t(34) = 2.59, p = .014, d = .44), while the change scores of those in the Uncoordinated condition did not (t(34) = 0.243, p = .810, d = .04). See Table 4 for the descriptive statistics for all attitude measures.

**Table 4:** Descriptive statistics for the attitude change scores.

		Mean (SD)	Median (range)
Comparison Attitudes	Synchronous	-3.83(8.73)	-2.2 (-22.2–11.0)
	Uncoordinated	0.24 (5.85)	1.2 (-10.6–12.0)
Hungarian Attitudes	Synchronous	-0.48 (5.42)	0.09 (-9.73–15.73)
	Uncoordinated	-0.89 (6.75)	-1.64 (-13.55–20.18)
Roma Attitudes	Synchronous	-4.42 (7.91)	-2.82 (-20.27–10.18)
	Uncoordinated	-0.22 (8.36)	-1.97 (-12.64–17.09)

**Fig 3:**
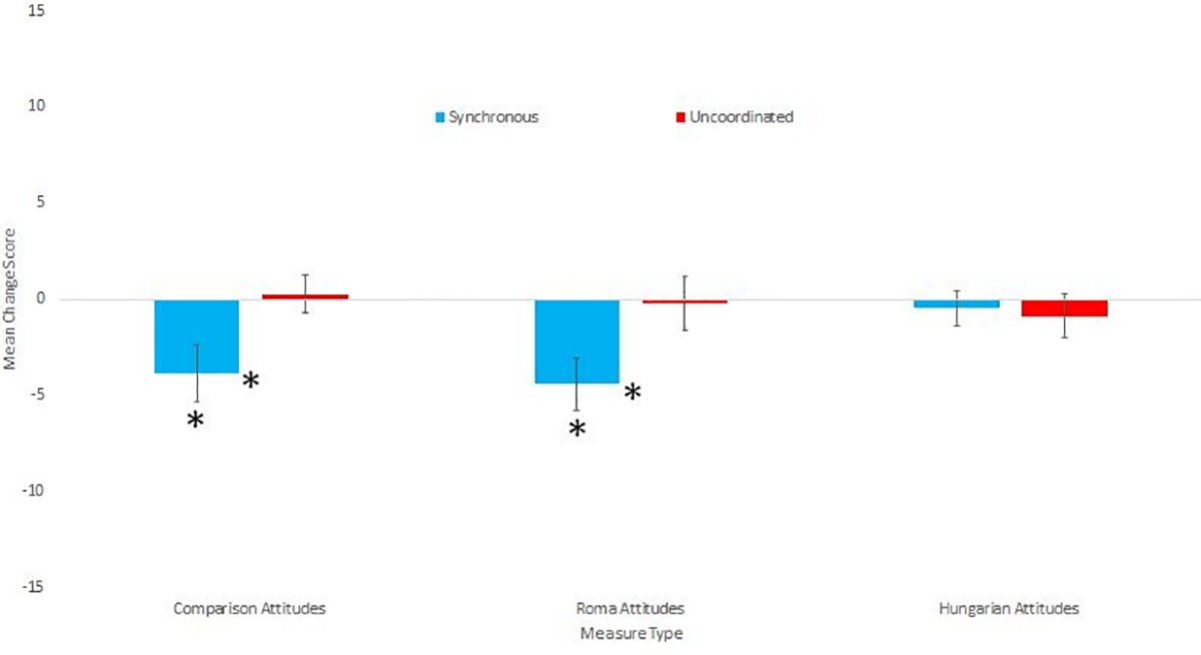
Means and standard errors for the attitude change scores.

Cronbach’s alphas for the Roma attitude measure was acceptable (a = .852), but the alpha for the Hungarian attitudes was not (a = .405). Removing items did not raise the alpha to an acceptable level. Because we were primarily interested in the Roma attitude scale, and used the Hungarian attitudes as a comparison, we combined the items into two composite change scores in the planned way. There was no significant difference between conditions for Non-Roma Hungarian attitude change scores (t(68) = -0.282, p = .779, d = .07), but those in the Synchronous condition had greater negative change scores (indicating less prejudice) than those in the Uncoordinated condition (t(68) = 2.157, p = .035, d = .51). We then analysed which of the Roma attitude change scores differed from 0 separately for each condition. Only the change scores of those in the Synchronous condition significantly differed from 0 (t(34) = 3.30, p = .002, d = 0.56), while the change scores of those in the Uncoordinated condition did not (t(34) = 0.156, p = .877, d = .03). Please see Table 4 for the descriptive statistics for all attitude measures. Fig 3 shows the mean and standard errors for the attitude measures.

### Implicit attitudes

Two d scores were calculated for each participant (pre and post); d is essentially a variant of Cohen’s d [53]. The resulting score, d, is a measure of response time differences ranging from -2 to + 2, which tells us the direction and the strength of any relationship between the targets and attribute categories. The closer a score is to -2, the stronger the association between Roma/Good and Hungarian/Bad. Conversely, the closer a score is to +2, the stronger the association between Roma/Bad and Hungarian/Good. A score of 0 shows there is no association whatsoever between the categories. To compute d (using the improved algorithm), and the resulting Cronbach’s alphas we used custom R code [49].

Any response times <400ms or >10000ms were excluded (exclusions = 2.1% of data). Individual block means were then calculated and any response times for incorrect trials were replaced with the block mean plus a penalty of 600ms. The mean of each experimental block was calculated and a difference score for blocks 3–6, and 4–7 was then computed. Each difference score was then divided by the pooled SD of each of the two blocks, and d is the average of these two resulting scores. Cronbach’s alphas confirmed the internal reliability of the d scores (Pre α = .85; Post α = .85), as all scores fell over the typical cut-off point of .75. This resulted in a Pre and Post d score for each individual; a change score was then calculated.

**Fig 4:**
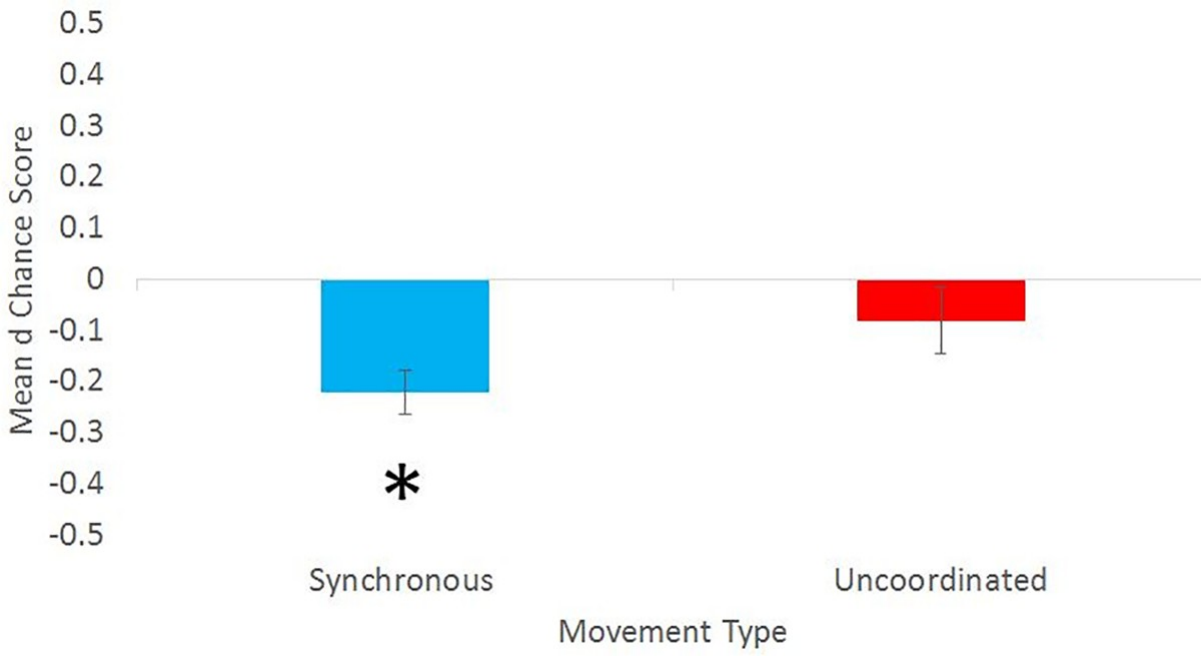
Means and standard errors for the d change scores.

Those in the Synchronous condition (m = -.22, sd = .314) had larger negative change scores (indicating a greater move towards less of a negative/Roma association) than those in the Uncoordinated condition (m = -.080 sd = .387) but this difference did not reach significance (t(58) = 1.66, p = .102, d = .40). In order to determine whether either IAT change score differed from 0 in either condition, we also used one sample t tests. The change scores of those in the synchronous condition significantly differed from 0 (t(29) = 4.151, p < .001, d = 0.57), while the change scores of those in the uncoordinated condition did not (t(29) = -1.23, p = .227, d = .25). Fig 4 shows the mean and standard errors for the d change scores.

## Discussion

After individuals had participated in synchronous walking with a Roma confederate they reported greater affiliation towards their partner. To our knowledge, this is the first research to show that motoric synchronicity can increase affiliation amongst people even when they are part of a socio-culturally significant out-group. It is worth noting that work on another form of interpersonal coordination, mimicry, has also been found to reduce attitudes in similar contexts [54, 55]. However, in this study individuals who walked in a synchronous way also saw increases in empathy towards the Roma group more generally. Interestingly, the participants did not show significant increases in overlap, neither towards their partner nor towards the Roma group. Instead, it appears that differentiation between the self and the Roma partner, and the Hungarian and Roma groups at large remained intact despite reductions in prejudice following coordination. This suggests that synchronicity’s pro-social effects are not simply the result of a blurring of representational overlap. This is in line with the model of mutual intergroup differentiation [56], in which the authors cite a need to maintain distinctions between two groups in order to generalize changes in positive attitudes towards an individual out-group member and towards an out-group as a whole. Other research also suggests that interventions that allow for preservation of in-group and out-group identities may be particularly effective for those who are strongly aligned with their in-group [57]. Thus, interventions such as this, which promote reductions in prejudice while maintaining inter-group differences, may be effective in political climates where in-group, nationalistic identities are particularly salient.

Those who walked synchronously showed a significant decrease in explicit negative attitudes towards Roma, and a decrease in how different they felt Roma people were compared to Non-Roma Hungarians. These participants also showed some evidence for decreased implicit negative attitudes, as demonstrated by the significant IAT change scores in the Synchronous but not Uncoordinated conditions (though it is worth noting that no significant differences in D change scores between conditions were observed). While both implicit and explicit attitudes are found to predict behaviour, working both separately or in tandem [58], they are thought to develop differently [59], operate through different mechanisms [60], and have distinct routes for change [61]. Specifically, it is conjectured that implicit attitudes reflect an automatic evaluative reaction in response to a relevant stimulus, while explicit attitudes arise through a more reflective process that relies on propositions (I dislike X) [61]. Thus, implicit attitudes are a reflection of the associations a person has been exposed to previously, while explicit attitudes are a reflection of the extent to which a person endorses these associations [62].

Using these principles, research has shown that implicit biases can be influenced through exposure to different associations, such as repeated pairing of an object and an attitude [62] or through an activation of alternative patterns, such as showing pictures of revered Black public figures and disliked White public figures [63]. This has been achieved by shifting the context in which outgroups are presented. For instance, implicit biases for outgroup members are reduced when they are categorized by occupation rather than race [64] or when pportrayed within positive (family barbecue) rather than negative (gang) settings [65] . For these reasons, it is perhaps unsurprising that there were no significant differences between Synchronous and Uncoordinated conditions with regards to IAT change scores, as research indicates that mere exposure to an out-group member can influence implicit biases [66, 67]. As both groups were exposed to the Roma confederate in a professional context (in a university lab), it follows that both conditions experienced a reduction in implicit bias. While changes in IAT scores being merely due to learning or repetition effects cannot be ruled out, it is of interest to note that only the synchronous group saw *significant* reductions in implicit bias, with a large effect size (d = .57). This suggests that aspects of synchronous movement in particular may have positively shifted the context in which the Roma group was represented.

Synchronous walking was also shown to cultivate changes in explicit attitudes. As both implicit and explicit changes in prejudice influence future behaviour towards out-group members [68], it appears that coordinated walking with an out-group member is an effective means towards creating positive attitudes towards out-groups. Though more simplistic and brief than other contact interventions, motoric synchrony has an advantage over other forms of intergroup contact as it does not rely on a common language or shared cultural reference points between participants. However, it can still suffer from many of the logistic and pragmatic barriers that make all contact interventions difficult, primarily that it can be difficult to get members of polarised groups in the same room.

For instance, it is common for minority groups to live in segregated areas [69], thus making real life contact with someone from another group challenging. Furthermore, friendship groups are particularly homogenous with regards to racial and ethnic similarities [37]; one study on a national probability sample showed only 8% of American adults having a person of another race with whom they are able to confide in, which is less than one/seventh the heterogeneity that would be observed if people chose at random from the population [70]. This suggests that not only logistic barriers, but social barriers tied to homophily, or the principle that contact naturally occurs more between similar versus dissimilar people, make implementation of contact interventions difficult.

For this reason, researchers have devised ways of utilizing inter-group contact paradigms through more indirect methods. One method is to have people simply imagine having social contact with an out-group member [71]. Some suggest that imagining social interactions with another individual creates many of the same pro-social effects as real life contact, while also reducing explicit and implicit biases [72], though to a somewhat lessened degree than real contact [73]. As previous research indicates that mentally simulated synchrony cultivates some of the same social bonding effects as actual synchronicity [42] it stands to reason that imagined synchrony may also be capable of reducing stereotyping akin to actual synchrony.

## Method–Study 2

Sixty people participated in this study (18 males and 42 females, M_age_ = 25.9yr, SD_age_ = 7.23). All participants were recruited form the Central European University’s database and consisted of both CEU students, students from other local universities, and non-student populations. Inclusion criteria were the same as for Study 1, with the addition that anybody who had taken part in Study 1 was excluded, which is why sample size was slightly reduced. All participants were naïve to the aims of the study and were compensated with a 1500 HUF (roughly 5 euros) gift voucher. The experiment was approved by the (EPKEB) United Ethical Review Board for Research in Psychology and took approximately 25 minutes to complete.

All materials and procedures were identical to Study 1, except this time participants did not actually meet a Roma person to walk with them, but were introduced to a confederate via a video recording. The confederate was introduced as a Roma participant ‘participating in an adjacent lab’. Each party recorded a greeting to introduce themselves to the other person using a primed introduction, in Hungarian (“Hi, my name is X, I am X years old, and I am the Roma/Non Roma Participant”). Pre-recorded introductions were used for the Roma individuals. This time a male and a female confederate were used in order to have same gender pairs. Both confederates were of a similar age (mid 20s), and from the Roma community. These video introductions were closely matched across conditions, and roughly equal amounts of each gender were assigned to each condition. Following this brief introductory video, participants were asked to simply imagine walking with the other participant using the below instructions. An extra check question was also added asking how successful participants thought they were at the imagination task, since it was not possible to have an objective measure of task success. Participants were first shown a short 10 second video of two people walking down an empty corridor in the required way in order to try and ensure people imagined the required interactions. Participants were then asked to close their eyes and spend two minutes imagining walking with the other person. Again, all measures and the following instructions were given in Hungarian:

### Experimental

“You are now going to perform an imagination task with the other participant. You will close your eyes and spend 2 minutes imagining walking like you will see in this video. Try to imagine both yourself and the other participant walking together in synchrony as vividly as you can. Imagine the sounds you would hear as each of your feet reverberate on the floor at the same time. Imagine looking around and seeing the other person walking in perfect time with you.”

### Control

“You are now going to perform an imagination task with the other participant. You will close your eyes and spend 2 minutes imagining walking like you will see in this video. Try to imagine both yourself and the other participant each walking at your own pace as vividly as you can. Imagine the different sounds you would hear as each of your feet reverberate on the floor at different times. Imagine looking around and seeing the other person walking at their own pace.”

## Results–Study 2

### Manipulation checks

We first checked that participants in each condition were performing the walking task as requested using the three check questions measuring task difficulty, success and perceived coordination. While those in the coordinated condition did report more perceived coordination than those in the uncoordinated condition (u = 719, p < .001, r = .51), neither task difficulty (u = 407.0, p = .525, r = .08) nor task success (u = 468.0, p = .790, r = .03) significantly differed between the two conditions. See Table 5 for descriptive statistics.

**Table 5:** Descriptive statistics for the manipulation check questions in Study 2.

		Mean (SD)	Median (range)
Perceived Coordination	Synchronous	78.43 (18.89)	83.5 (38–100)
	Uncoordinated	52.0 (24.52)	46 .67 (10–100)
Task Difficulty	Synchronous	33.8 (26.69)	28.0 (0.0–92.0)
	Uncoordinated	39.43 (29.61)	37.5 (0.0–90.0)
Task Success	Synchronous	72.53 (18.22)	76.0 (18–100)
	Uncoordinated	71.0 (22.98)	73.5 (20–100)

### Affiliation, overlap and empathy towards the confederate

**Fig 5:**
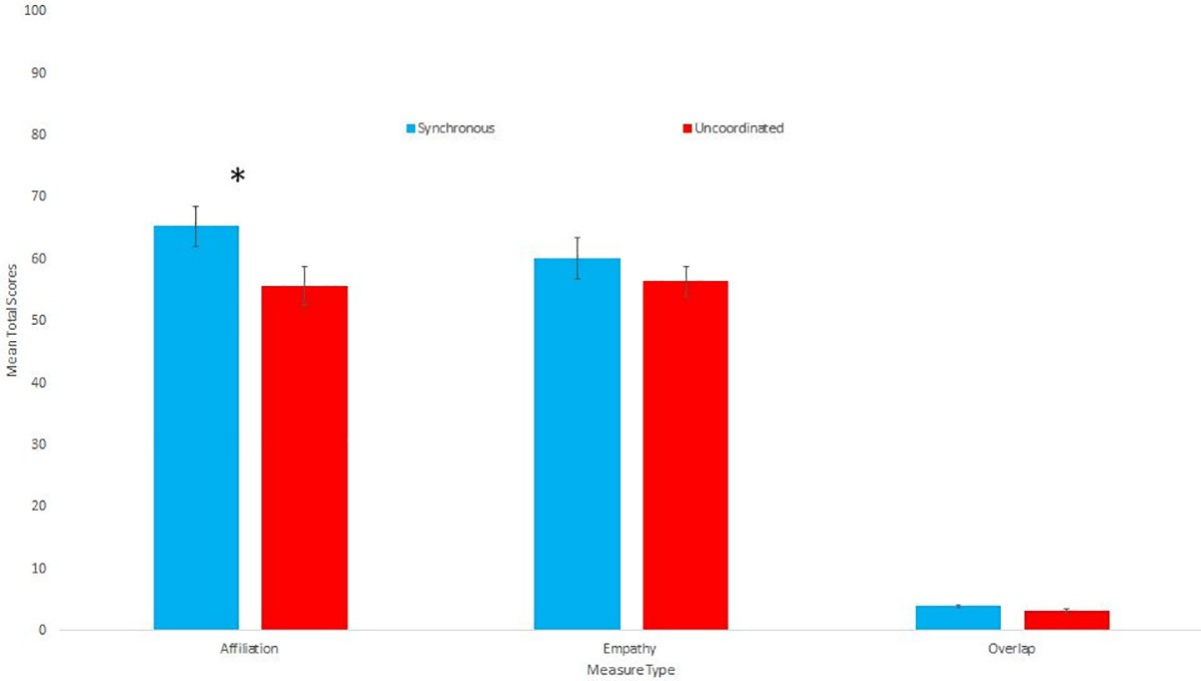
Means and standard errors for the Attitude change scores affiliation, empathy and overlap ratings towards the confederate in Study 2.

We then explored whether there was any difference in ratings of affiliation, overlap or empathy towards the confederate. We first combined the five affiliation items into a composite score by taking the mean (Cronbach’s a = .876). Those who had imagined walking in a Synchronous way reported feeling significantly more affiliated towards the confederate than those who had imagined walking in an Uncoordinated way (t(58) = 2.14, p = .037, d = 63). We also combined the five empathy items into a composite score by taking the mean (Cronbach’s a = .837). Those in the Synchronous condition reported more empathy towards the confederate than those in the Uncoordinated condition, but this difference was not significant (u = 539.0, p = .188 r = 0.13). While on average those in the coordinated condition reported more overlap towards the confederate than those in the uncoordinated condition, this difference was not quite significant at the .05 level (t(58) = 2.0, p = .05, d = .52). Table 6 gives the descriptive statistics for all of the above measures. Fig 5 shows the mean and standard errors.

**Table 6:** Descriptive statistics for the total affiliation, empathy and overlap ratings towards the confederate in Study 2.

		Mean (SD)	Median (range)
Affiliation	Synchronous	65.28 (17.8)	64.1 (19.2–98.6)
	Uncoordinated	55.61 (17.23)	55.6 (21.6–100)
Empathy	Synchronous	60.1 (17.74)	60.6 (13.4–94.8)
	Uncoordinated	56.43 (13.29)	55.0 (38.6–96.0)
Overlap	Synchronous	3.97 (1.5)	3.93 (1–7)
	Uncoordinated	3.23 (1.33)	3.13 (1–6)

### Empathy and overlap towards Hungarian vs Roma groups

**Fig 6:**
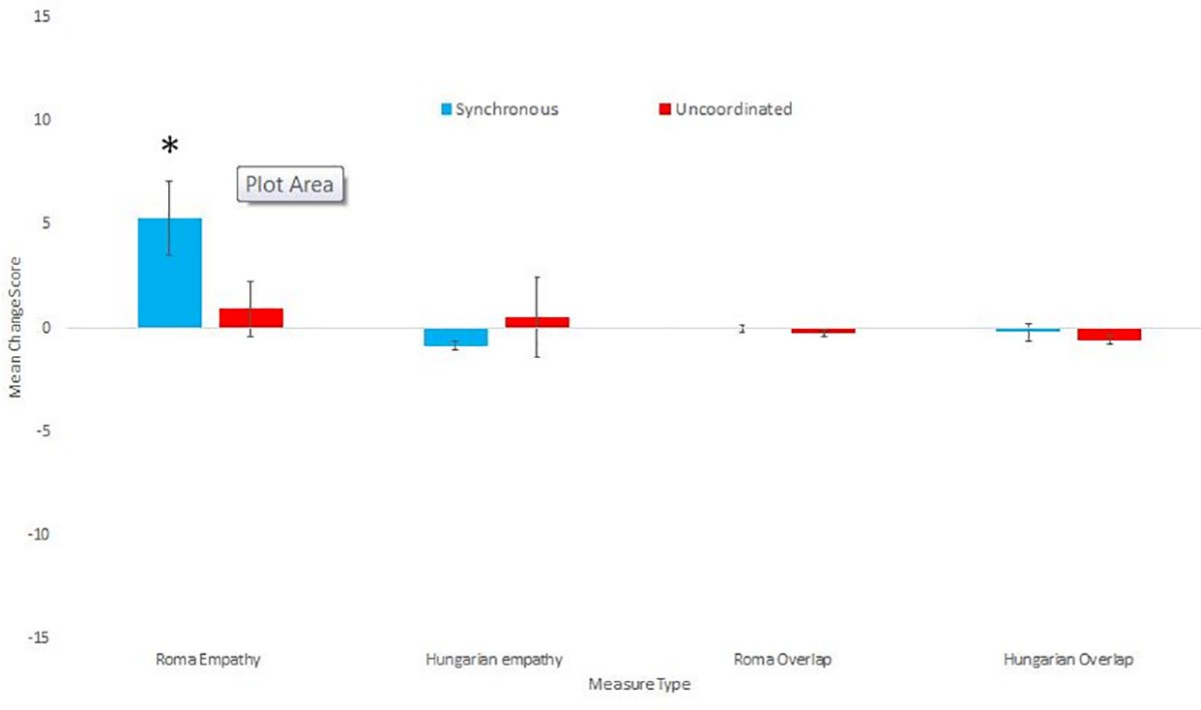
Means and standard errors for Empathy and Overlap change scores towards each group type in Study 2.

There was no significant difference in overlap change scores between the conditions for either Roma overlap (u = .495.5, p = .457 r = 0.1) or Non-Roma Hungarian overlap (u = 538.0, p = .177 r = 0.17). For empathy scores, (Roma empathy a = .88, Non-Roma Hungarian a = .879), there was no significant difference in change scores between the conditions for either Roma empathy (u = 546, p = .156 r = 0.183) or Non-Roma Hungarian empathy (u = .449.5, p = .994 r <0.001). As in Study 1, we also explored whether any of these change scores differed from 0 separately for each condition. In line with Study 1, only the Roma empathy change score in the Synchronous condition significantly differed from 0 (Z = 360.5, p = .008, r = 0.48), while none of the Synchronous Non-Roma Hungarian empathy (t(29) = -.5280, p = .602, d = .096), Uncoordinated Roma empathy (t(29) = -.680, p = .502, d = .125), or the Uncoordinated Non-Roma Hungarian empathy (Z = 223.5, p = .853, r = 0.04) change scores differed significantly from 0. All descriptive statistics for the overlap and empathy measures can be found in Table 7. Fig 6 shows the mean and standard errors.

**Table 7:** Shows the descriptive statistics for Empathy and Overlap change scores towards each group type in Study 2.

		Mean (SD)	Median (range)
Roma Overlap	Synchronous	-0.07 (0.64)	-0.08 (-1.0–1.0)
	Uncoordinated	-0.27 (1.02)	-0.23 (-3.0–2.0)
Hungarian Overlap	Synchronous	-0.23 (1.19)	-0.17 (-3.0–2.0)
	Uncoordinated	-0.60 (1.04)	-0.6 (-3.0–1.0)
Roma Empathy	Synchronous	5.27 (9.83)	4.2 (-7.8–35.8)
	Uncoordinated	0.91 (7.3)	1.0 (-17.2–14.0)
Hungarian Empathy	Synchronous	0.87 (9.06)	-0.3 (-22.6–19.0)
	Uncoordinated	-0.49 (10.59)	-2.0 (-12.4–26.4)

### Explicit attitudes towards Hungarian vs Roma groups

**Fig 7:**
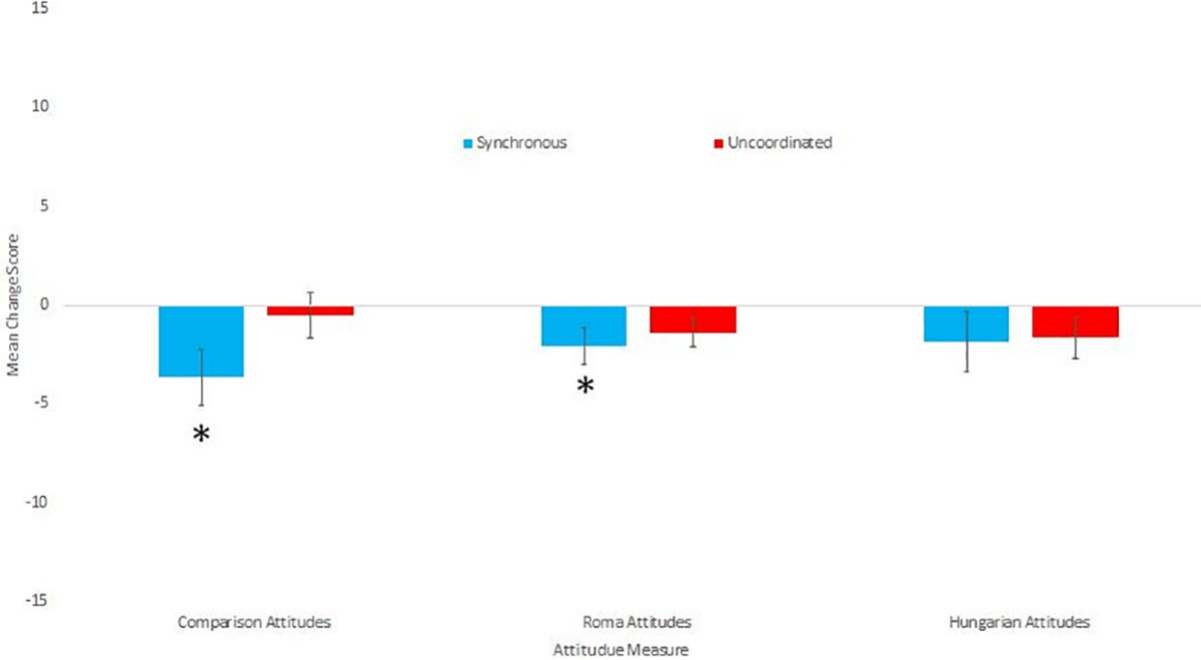
Means and standard errors for Attitude change scores for Study 2.

While those in the Synchronous condition did report smaller comparison change scores (a = .678) than those in the uncoordinated condition, this was not significant (t(58) = 1.714, p = .092, d = .44). As in Study 1, we also explored whether either of these change scores differed from 0 separately for each condition. Only the change scores of those in the Synchronous condition significantly differed from 0 (t(29) = 2.594, p = .015, d = 0.47), while the change scores of those in the Uncoordinated condition did not (t(29) = 0.439, p = .664, d = .07). Cronbach’s alphas for the Hungarian attitudes was .627, and for the Roma attitudes .891. There was no significant difference between conditions for Non-Roma Hungarian (t(58) = .110, p = .913, d = .03), or Roma (t(58) = .548, p = .586, d = .14) change scores. As in Study 1, we also explored whether Roma attitude change scores differed from 0. The change scores of those in the Synchronous condition significantly differed from 0 (t(29) = 2.171, p = .038, d = 0.56), while the change scores of those in the Uncoordinated condition did not (t(29) = 1.863, p = .073, d = .03). See Table 8 for the descriptive statistics for all attitude measures. Fig 7 shows the mean and standard errors.

**Table 8:** Descriptive statistics for the all Attitude change scores for Study 2.

		Mean (SD)	Median (range)
Comparison Attitudes	Synchronous	-3.65 (7.70)	-2.7 (-25.4–10.0)
	Uncoordinated	-0.51 (6.40)	-0.93 (-10.6–11.2.)
Hungarian Attitudes	Synchronous	-1.85 (8.41)	-1.27 (-22.91–16.91)
	Uncoordinated	-1.65 (5.68)	-1.5 (-16.82–8.55)
Roma Attitudes	Synchronous	-2.05 (5.18)	-2.36 (-9.55–14.09)
	Uncoordinated	-1.39 (4.10)	-0.5 (-9.73–5.18)

### Implicit attitudes

**Fig 8:**
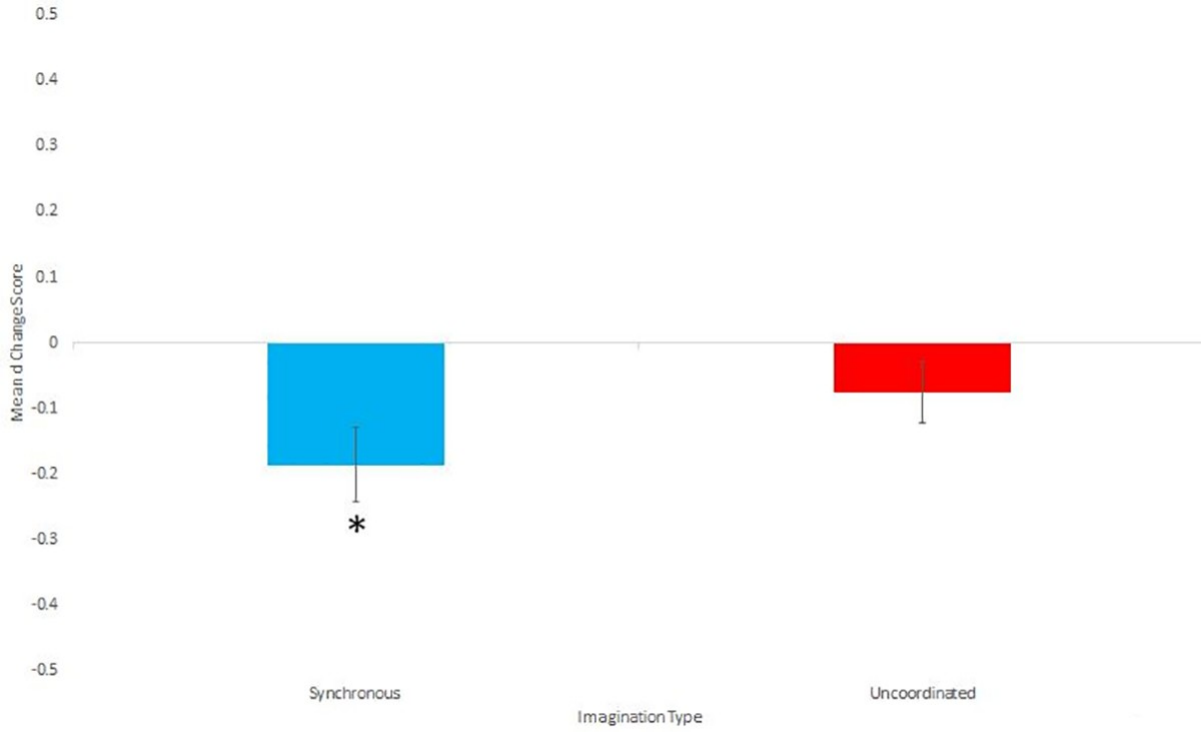
Means and standard errors for the d change scores for study 2.

IAT data was scored in exactly the same way as described in Study 1. This time 1.9% of total response times were excluded for being <400ms and >10000ms. Cronbach’s alphas were acceptable (Pre, α = 78; Post, α = .81). Those in the Synchronous condition (m = .-185, sd = .306) had larger negative change scores than those in the Uncoordinated condition (m = -.0754, sd = .255) but this was not significant (t(58) = 1.51, p = .137, d = .39). In order to determine whether either IAT change scores differed from 0 we also used one sample t-tests. The change scores of those in the synchronous condition significantly differed from 0 (t(29) = 3.318, p = .002, d = .60), while the change scores of those in the Uncoordinated condition did not (t(29) = 1.617, p = .117, d = .29). Fig 8 shows the mean and standard errors for the d change scores.

## Discussion–Study 2

Imagined walking produced some of the same effects as actual walking with regards to improved attitudes towards the Roma partner and the Roma group in general. Specifically, imagined synchrony led to more affiliation towards the partner, and a reduction in both explicit prejudice and implicit attitudes, in line with Study 1 (though again it is worth noting that while d change scores were significant, between group differences were not). Effect sizes were quite similar, suggesting that imagined rather than actual walking did not dilute the effect of synchrony. This may be attributable to the fact that participants were given detailed, elaborate instructions of what synchronous walking entails; they were shown a video and given a long verbal summary. Furthermore, they were not simply told to imagine a Roma person from memory but viewed a video introduction from a Roma person. Both these additions are in line with the type of elaboration that researchers have found to lead to enhanced imagined contact effects [74].

There were however a few deviations from Study 1’s results. For one, it is worth noting that unlike in Study 1 we did not observe significant between group differences in group level measures between the Synchronous and Uncoordinated conditions. Change scores however indicated that significant changes from 0 were only seen in the Synchronous condition for group level measures. This suggests that while imagined synchronous walking is having an effect on attitude measures, (as indicated by change score analysis), this was not reliably different from imagined asynchronous walking (as indicated by between group analyses). There are multiple reasons why this may be the case. For one, despite giving detailed instructions of what was to be imagined, we have no way of knowing for sure what a participant actually imagined, though self-report scores did confirm there was more imagined coordination in the relevant condition. It may be the case that simply imagining walking with a person of a minority group may be enough to reduce prejudice towards them to some (albeit a weaker) extent. Indeed, some evidence suggests that mere exposure to an out-group member can reduce social distances with this group [66, 67]. However, it was only in the imagined synchrony condition that we saw significant decreases in negative attitudes.

It is also worth noting that following imagined synchronous walking participants reported greater overlap with their imagined co-actor (though this was not quite significant at the .05 level). Conversely, imagined walking did not appear to influence empathy towards the co-actor, though it did increase empathy towards the larger Roma group. The first of these findings may be related to the combined sensory deprivation and attentional demands placed on the individual by the imagination task. For example, in the ‘rubber hand illusion,’ participants perceive a rubber hand as belonging to themselves when the proprioceptive and visual sensations of the rubber hand match the hidden, tactile perceptions they experience when simultaneously touched [75]. Unlike in the rubber hand illusion, in the imagined condition participants do not have any direct sensory experiences of the other. However, they are primed to visualize that another person has identical movements, and that this person is beside them. Balancing the visualization of both the self and partner may have led to participants blurring the self and the other and perhaps conflating agency of the movements. As individuals in the walking condition had visual, tactile and proprioceptive information available throughout the task, this may be why there was overlap in the imagined rather than actual synchrony task. This may also explain why there were no increases in empathy following the imagined condition; participants and co-actors were perhaps less individuated and thus less likely to view the co-actor as an empathetic, separable agent.

In conclusion, it appears that imagined synchronous walking may be capable of producing some of the same effects as actual synchronous walking. As there are many pragmatic barriers that make real life contact difficult, being able to maintain these effects without necessitating the physical presence of a person from another group makes this a noteworthy addition to contact interventions.

## Additional analyses and general discussion

In order to compare findings across Study 1 and 2 we undertook additional exploratory analyses comparing actual to imagined interactions for all of the analyses reported above using Univariate ANOVAs. All descriptive statistics can be found in the tables previously reported.

For affiliation towards the confederate the only main effect was Condition (F(1,126) = 10.99, p = .001), the main effect of Experiment was not quite significant (F(1,126) = 3.77, p = .054) and there was no interaction (F(1,126) = .007, p = .935). For overlap towards the confederate the only significant main effect was Condition (F(1,126) = 5.72, p = .018), neither the main effect of Experiment (F(1,126) = 0.034, p = .853) nor the interaction (F(1,126) = .614, p = .435) were significant. For empathy towards the confederate the only significant main effect was Condition (F(1,126) = 4.272, p = .041), neither the main effect of Experiment (F(1,126) = 1.345, p = .248) nor the interaction (F(1,126) = .387, p = .535) were significant.

For overlap with the Roma group the main effects of Condition (F(1,126) = 0.987, p = .322), Experiment (F(1,126) = 2.258, p = .135) and the interaction (F(1,126) = .006, p = .939) were not significant. For overlap towards Hungarians the main effect of Condition (F(1,126) = 3.82, p = .053) was not quite significant and neither the main effect of Experiment (F(1,126) = 0.001, p = .991) nor the interaction (F(1,126) = .075, p = .785) were significant. For empathy towards the Roma group the only significant main effect was Condition (F(1,126) = 10.31, p = .002), neither the main effect of Experiment (F(1,126) = 1.414, p = .237) nor the interaction (F(1,126) = .898, p = .345) were significant. For empathy towards Hungarians the main effect of Condition (F(1,126) = 0.186, p = .667), Experiment (F(1,126) = .269, p = .605) and the interaction (F(1,126) = 1.123, p = .291) were not significant.

For comparison attitudes the only significant main effect was Condition (F(1,126) = 7.922, p = .006), neither the main effect of Experiment (F(1,126) = 0.05, p = .824) nor the interaction (F(1,126) = .134, p = .715) were significant. For direct attitudes towards the Roma only the main effect of Condition (F(1,126) = 4.162, p = .043) was significant, the main effect of Experiment (F(1,126) = 0.25, p = .618) and the interaction (F(1,126) = 2.206, p = .140) were not significant. For direct attitudes towards Hungarians the main effect of Condition (F(1,126) = 0.008, p = .928), Experiment (F(1,126) = 0.836, p = .362) and the interaction (F(1,126) = .07, p = .792) were not significant. For implicit attitudes the only significant main effect was Condition (F(1,126) = 4.86, p = .029), the main effect of Experiment (F(1,126) = .125, p = .725) and the interaction (F(1,126) = .070, p = .729) were not significant.

Taken together, Study 1 and Study 2 demonstrate that walking in synchrony and imagining walking in synchrony with an out-group member can increase feelings of affiliation towards a specific out-group partner, as well as increasing empathy towards the out-group as a whole and reducing explicit and implicit negative attitudes. Our findings add several new dimensions to both the coordination literature as well as research on prejudice reduction. First, coordination’s pro-social effects towards out-group members can be demonstrated using culturally and societally relevant groups rather than those created through a minimal-group design. This means that this is an intervention that could be applied in real-world settings in an effort to tackle prejudice between disparate cultural groups. It is worth noting that the follow up measures were taken shortly after the walking manipulation, so is it unclear how transient these changes are. Future work should aim to explore the longevity of the effects. Secondly, this work suggests that these effects cannot be attributed to a generalised increase in pro-sociality post coordination (as has been suggested by [44]) but generalise specifically to the interaction partner’s social group (as has been suggested by [76]). Although not supported by our data, the interpretation that such effects are generalised cannot be ruled out entirely, and future work should address this by testing attitudes towards in as well as outgroup members following embodied interventions with individuals from those groups.

Lastly, our study provides some evidence for implicit changes following coordination, extending prior work that has shown a reduction in implicit negative attitudes following experienced ownership over an out-group hand [26, 27]. Whether distinct processes underlie change of implicit attitudes through behavioural synchronization and experienced ownership is an important topic for future research. More generally, affecting change in implicit bias is important, as research indicates that within interactions between groups, implicit attitudes drive perceived friendliness towards an out-group member [68]. As individuals have considerably less conscious control over their implicit biases [77], and these biases are automatically expressed when an individual is under pressure [78], developing interventions that affect more automatic tendencies may allow people to reduce biases towards non-group members that they were not aware they possessed. As research has shown that both explicit prejudice and implicit forms of prejudice stereotyping influence a person’s behaviour towards out-group members [68], providing interventions that alter both types of biases may be particularly effective with regards to external validity. Thus, improvements on both implicit and explicit measures following interpersonal coordination indicate that inducing synchrony may be an effective intervention for reducing prejudice and influencing positive actions towards members of out-group communities.
