# Correction: Imagine All The Synchrony: The effects of actual and imagined synchronous walking on attitudes towards marginalised groups

**DOI:** 10.1371/journal.pone.0220264

**Published:** 2019-07-18

**Authors:** The PLOS ONE Staff

In the Introduction, there is an error in the last sentence of the first paragraph; due to a typesetting error, the word “acceptable” appears as “accep1”. The publisher apologizes for this error. The complete, correct sentence is: When negative stereotypes about a certain group become pervasive in society, it may be more acceptable for those in the majority to express overt prejudice towards minority groups, leading to persecutions [1].

As a result of the typesetting process, the placement of Figs 1–8 and Tables 2–8 in the PDF version of the article differs from the XML version of the published article. The publisher apologizes for this difference in content placement.

In the Discussion section, there is an error in the second sentence of the second paragraph; the “D change scores” should not include a capital “D”. The correct sentence is: These participants also showed some evidence for decreased implicit negative attitudes, as demonstrated by the significant IAT change scores in the Synchronous but not Uncoordinated conditions (though it is worth noting that no significant differences in d change scores between conditions were observed).

In addition, there is an error in the second sentence of the third paragraph of the Discussion, in which “portrayed” appears as “pportrayed.” The correct sentence is: For instance, implicit biases for outgroup members are reduced when they are categorized by occupation rather than race [64] or when portrayed within positive (family barbecue) rather than negative (gang) settings [65].

In Tables 2 and 6, an extra grid line is included in the first column of the “Overlap” row. Please see the correct Table 2 and Table 6 here.

**Table 2:** Descriptive statistics for the total Affiliation, Empathy and Overlap ratings towards the confederate.

		Mean (SD)	Median (range)
Affiliation	Synchronous	70.58 (13.74)	68.0 (43.0–100)
	Uncoordinated	61.37 (16.07)	62.4 (10.2–95.2)
Empathy	Synchronous	64.63 (14.32)	63.0 (39.2–100)
	Uncoordinated	57.79 (12.23)	58.4 (17.0.– 79.6)
Overlap	Synchronous	3.74 (1.34)	3.71 (2–7)
	Uncoordinated	3.37 (1.09)	3.32 (2–5)

**Table 6:** Descriptive statistics for the total affiliation, empathy and overlap ratings towards the confederate in Study 2.

		Mean (SD)	Median (range)
Affiliation	Synchronous	65.28 (17.8)	64.1 (19.2–98.6)
	Uncoordinated	55.61 (17.23)	55.6 (21.6–100)
Empathy	Synchronous	60.1 (17.74)	60.6 (13.4–94.8)
	Uncoordinated	56.43 (13.29)	55.0 (38.6–96.0)
Overlap	Synchronous	3.97 (1.5)	3.93 (1–7)
	Uncoordinated	3.23 (1.33)	3.13 (1–6)

There are errors in references 22, 59, 61, and 76. Please see the correct references here:

22. Blascovich, J., Mendes, W. B., Hunter, S. B., Lickel, B., & Kowai-Bell, N. (2001). Perceiver threat in social interactions with stigmatized others. *Journal of personality and social psychology*, *80*(2), 253. http://doi.org/10.1037/0022-3514.80.2.253

59. Ranganath, K. A., & Nosek, B. A. (2008). Implicit attitude generalization occurs immediately; explicit attitude generalization takes time. *Psychological Science*, *19*(3), 249–254. https://doi.org/10.1111/j.1467-9280.2008.02076.x

61. Gawronski, B., & Bodenhausen, G. V. (2006). Associative and propositional processes in evaluation: an integrative review of implicit and explicit attitude change. *Psychological bulletin*, *132*(5), 692. http://doi.org/10.1037/0033-2909.132.5.692

76. Cirelli, L. K., Wan, S. J., & Trainor, L. J. (2016). Social effects of movement synchrony: increased infant helpfulness only transfers to affiliates of synchronously moving partners. *Infancy*, *21*(6), 807–821. https://doi.org/10.1111/infa.12140
